# Surgical approach strategies for open reduction internal fixation of closed complex tibial Pilon fractures based on axial CT scans

**DOI:** 10.1186/s13018-020-01770-y

**Published:** 2020-07-27

**Authors:** Yu Zhao, Jian Wu, Shijun Wei, Feng Xu, Changwang Kong, Xiaosong Zhi, Ming Huang, Xianhua Cai

**Affiliations:** 1 Department of Orthopaedic Trauma, First Affiliated Hospital of Dali University, Dali, People’s Republic of China; 2 Department of Orthopaedics, General Hospital of Central Theater Command (Wuhan General Hospital of Guangzhou Command, previously), NO. 627, Wuluo Road, Wuhan, 430030 Hubei Province People’s Republic of China; 3 Department of Orthopaedics, Changxing People’s Hospital, Changxing, Zhejiang Province, People’s Republic of China

**Keywords:** Pilon fracture, Distal tibial fracture, Surgical approach, Surgical strategy

## Abstract

**Background:**

To explore the application and clinical efficacy of surgical approach strategies in open reduction internal fixation of closed complex tibial Pilon fractures based on axial CT scans.

**Methods:**

This retrospective cohort study included data of 25 patients with closed complex tibial Pilon fractures treated from October 2011 to March 2014, including 19 males and 6 females aged 18–54 years (average 39.5 years). According to classification criteria of the Association for Osteosynthesis/Orthopedic Trauma Association (AO/OTA), 4 patients were type 43C1, 10 type 43C2, and 11 type 43C3. Surgical approaches were selected based on fracture line distribution and bone displacement revealed by axial CT scans, and an open reduction method was adopted for internal fixation of the bone plates. Postoperatively, Burwell-Charnley radiographic criteria were used to determine fracture reduction quality. Functional evaluation was performed using the American Orthopedic Foot and Ankle Society (AOFAS) Ankle-Hindfoot Scale. Complications, fracture union time, and the AOFAS scores at last follow-up were recorded.

**Results:**

The 25 included patients were followed for 22–60 months postoperatively (average follow-up 33.9 months). Of these, 19 patients achieved anatomical reduction of the articular surface, 5 achieved good reduction, and one achieved fair reduction. Two patients developed superficial infection on the anteromedial incision and delayed union but recovered well after local dressing change and oral administration of antibiotics. Another patient developed deep infection on the anterolateral incision, which was controlled by debridement, catheter irrigation, and intravenous antibiotic injection. All fractures healed well and average union time was 2.8 months (range, 2–3 months). No fracture malunion or internal fixation failures were found at last follow-up. All 25 patients had AOFAS scores ranging from 80 to 100 at last follow-up (average 88.4). Overall, 15 patients were excellent, 10 good, and 0 fair or poor, with excellent and good rates of 100%.

**Conclusions:**

Surgical approach strategies for complex tibial Pilon fractures based on axial CT scans accurately reconstruct the articular surface and achieve solid internal fixation of assembled locking plates, while early postoperative functional exercises contribute to the functional recovery of affected limbs and reduce related complications.

## Introduction

Tibial Pilon fractures are not common, accounting for only 5–7% of tibial fractures. Among all Pilon fractures, about 30% are complex Pilon fractures (AO/OTA43C type) caused by high-energy injuries. This type of fracture is often accompanied by severe soft tissue damage and is therefore characterized by high disability and teratogenicity as well as challenging treatment options [1].

The Association for Osteosynthesis/Association for the Study of Internal Fixation (AO/ASIF) first proposed four classic management principles for addressing tibial Pilon fractures, including (1) restoration of fibular length, (2) anatomic reduction of the articular surface, (3) filling the residual bone defect with cancellous autograft, and (4) stabilization of the medial column [2]. AO/ASIF also described classic surgical approaches for tibial Pilon fractures, including the anteromedial approach and the posterolateral approach, which are still widely used in clinical practice. In 2007, Assal et al. [3] modified the anteromedial approach to increase fracture exposure and decrease soft tissue-related complications. In 2011, the American Academy of Orthopedic Surgeons (AAOS) summarized six major surgical approaches for tibial Pilon fractures, namely, medial, anteromedial, anterolateral, lateral, posterolateral, and posteromedial, each of which has its own characteristics and range of exposure [4]. Cole et al. [5] studied the fracture line distribution and the position of main fracture fragments in 38 cases with comminuted AO/OTA 43-C3 tibial Pilon fractures, and concluded that a good understanding of fracture morphologies is conducive to the reasonable selection of surgical approaches. However, that study failed to offer detailed explanations about how to select a surgical approach based on the specific distribution of fracture lines and bone fragments. To date, it remains controversial as to whether a combined surgical approach has advantages over conventional surgical approaches when it comes to complex tibial Pilon fractures [6]. In addition, research is limited regarding the selection of strategies for surgical approaches for complex tibial Pilon fractures, and how to select a surgical approach for these fractures based on relatively objective indicators remains to be addressed.

According to the fracture line distribution and fracture fragment displacement revealed by axial CT scans, and in combination with local soft tissue conditions, this study aimed to select appropriate surgical approaches for complex tibial Pilon fractures (AO/OTA-43 C2, C3) and summarize the information on fracture reduction quality, the occurrence of related complications, and short-term efficacy of surgery. The objective of this study was to explore the application and clinical efficacy of surgical approaches strategies in open reduction and internal fixation of closed complex tibial Pilon fractures based on axial CT scans.

## Patients and methods

### Study purpose and design

This retrospective cohort study examined the application and clinical efficacy of surgical approach strategies in open reduction internal fixation of closed complex tibial Pilon fractures based on axial CT scans. The Internal Review Board of our institution reviewed and approved the study protocol. Because hospital records of the included patients were deidentified, signed informed consent for participation in the study was waived.

### Patients

A total of 25 patients with closed complex tibial Pilon fractures admitted to our institution from October 2011 to March 2014 were included in this study. Inclusion criteria were as follows: (1) diagnosis with closed tibial Pilon fractures and (2) falling into AO-OTA type 43 C1, C2, or C3. Exclusion criteria were as follows: (1) failure to meet the diagnostic and inclusion criteria; (2) having severe multiple injuries in brain, chest, or abdomen; (3) having severe internal morbidities or complications such as diabetes, hypertension, and heart disease; and (4) severe open tibial Pilon fractures.

Among the 25 included cases, all had closed complex tibial Pilon fractures, including 19 males and 6 females aged 18–54 years (average age 39.5 years). Causes of fractures were falling (19 patients), traffic accidents (3 patients), and industrial accidents (3 patients). According to AO/OTA classification standards, 4 cases were type 43C1, 10 were type 43C2, and 11 were type 43C3 (Table S1). Fractures at other sites were present in 8 patients, including right tibia plateau fracture, right femur fracture/left talus and calcaneal fracture, right calcaneal fracture, kidney injury/rib fracture, right tibial fracture, left calcaneal fracture, left patella fracture, and right tibia fracture/pelvic fracture (Table S2). Preoperative treatments included temporary fixation with external brackets, or calcaneal traction with traction weight of 4 kg followed by open reduction/internal fixation surgery after the swelling of the limbs subsided significantly and skin wrinkles appeared. The surgery times ranged from 10 to 25 days after injury (average 15.96 days). All patients underwent X-rays of the ankle joint and full length of the tibia and fibula in anteroposterior and lateral positions. Also, CT scans and three-dimensional reconstruction were performed to evaluate fracture line distribution and displacement of major fracture fragments.

### Formulation of surgical approaches

**Fig. 1:**
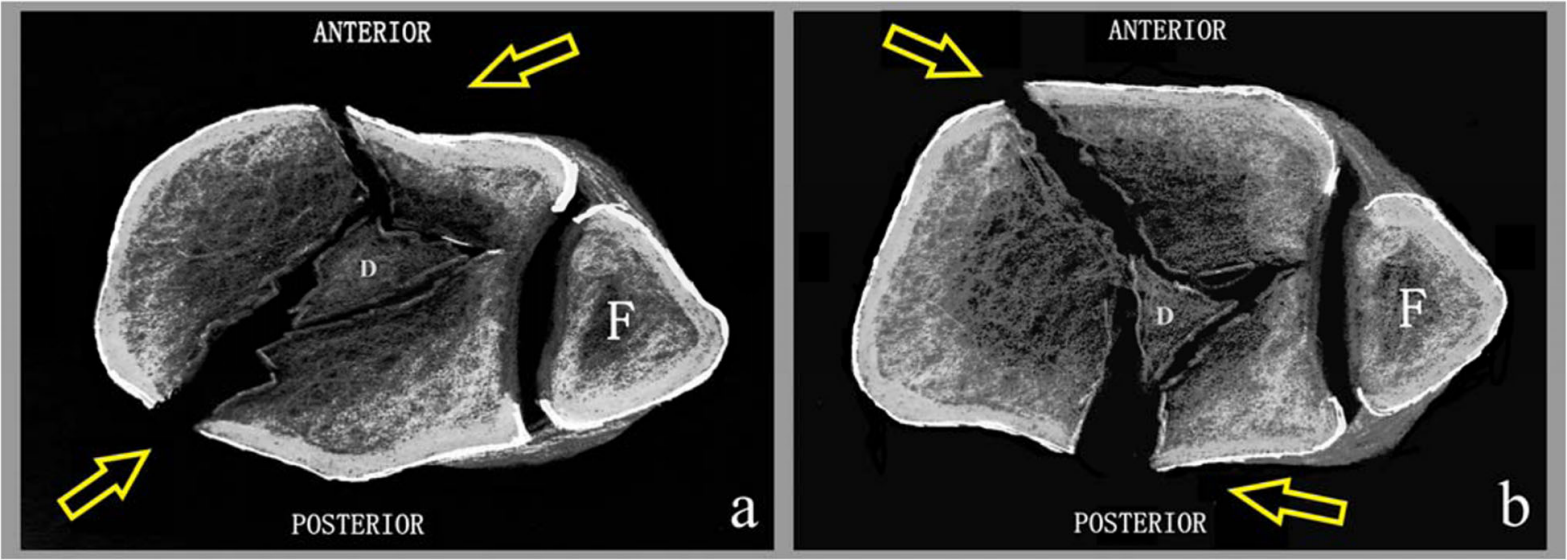
The surgical approach is established according to the fracture line and the displacement of the fracture fragments shown by CT scan 1 cm above the ankle joint surface. **a** The posterior malleolar fragment involves the tarsal tunnel, and the die-punch fragment is located laterally, using the posteromedial approach combined with the anterolateral approach. **b** The posterior malleolar fragment does not involve the tarsal tunnel, and the die-punch fragment is located medially, using the posterolateral approach combined with the anteromedial approach. The anterior approach is adjusted according to the position of the die-punch fragment and fracture line.

According to the fracture line distribution and fracture fragment displacement of the patients as displayed by axial CT scans, corresponding surgical approaches were developed. Specifically, when posterior malleolar fracture fragments did not affect the tarsal tunnel, a posterolateral approach combined with an anteromedial or anterior approach was adopted; otherwise, a posteromedial approach combined with an anterolateral or anterior approach was adopted. The anterior approach was subject to adjustment toward the lateral or medial side according to the position of the die-punch fragment (Fig. 1). Also, a posteromedial approach was adopted for 7 patients with an anterolateral or anterior approach and a posterolateral approach combined with an anteromedial or anterior approach was adopted for the remaining 18 patients (Table S2).

### Surgical procedures

#### Anesthesia and position

Among all included patients, those complicated with lumbar vertebral fractures underwent general anesthesia, and the others underwent epidural anesthesia. For the patients who needed a posterolateral approach combined with an anteromedial or anterior approach, a lateral float position was adopted, although the lateral position changed to a supine position after fracture reduction and fixation of the fibula and posterior malleolus were performed. For patients who needed a posteromedial approach combined with an anterolateral or anterior approach, a supine position was adopted, including placement of a soft pillow underneath the hip on the uninjured side in order to increase the external rotation of the affected lower limb and thus facilitates exposure of the posteromedial side.

#### Posterolateral approach combined with an anteromedial or anterior approach

**Fig. 2:**
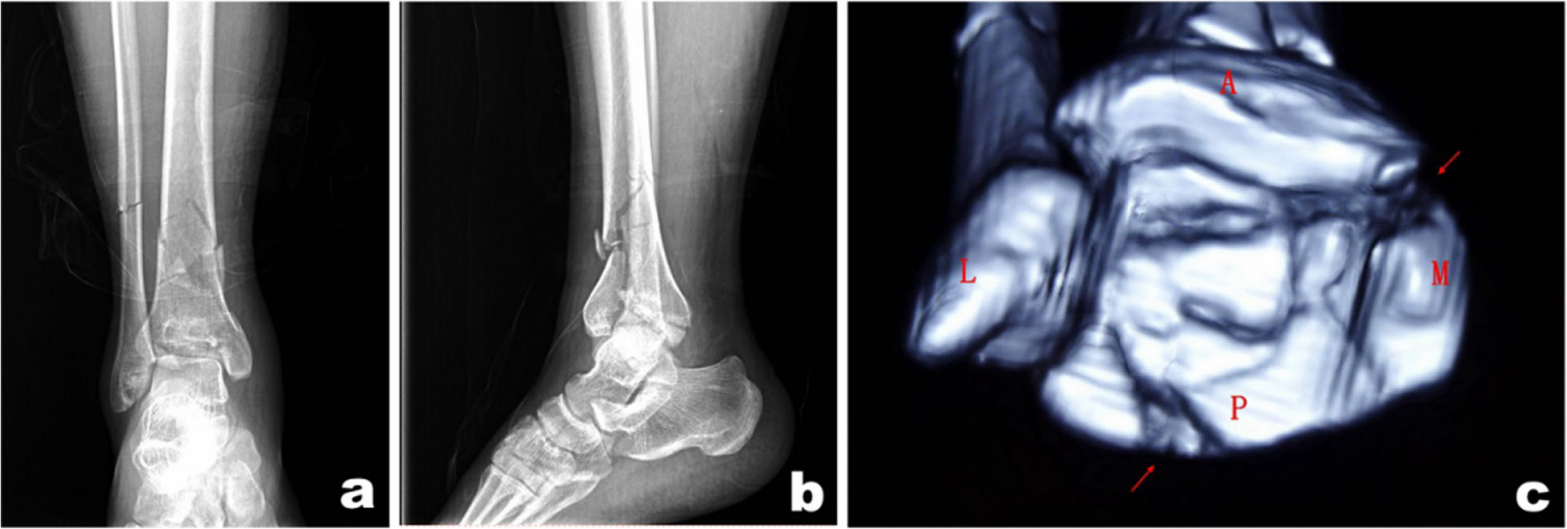
Male, 37 years old, right tibia pilon fracture caused by fall injury. Preoperative X-ray (**a**, **b**) and CT scan (**c**) showed collapse of the articular surface, the posterior malleolar fragment did not involve the tarsal tunnel area, and the die-punch fragment was slightly anterior and medial

**Fig. 3:**
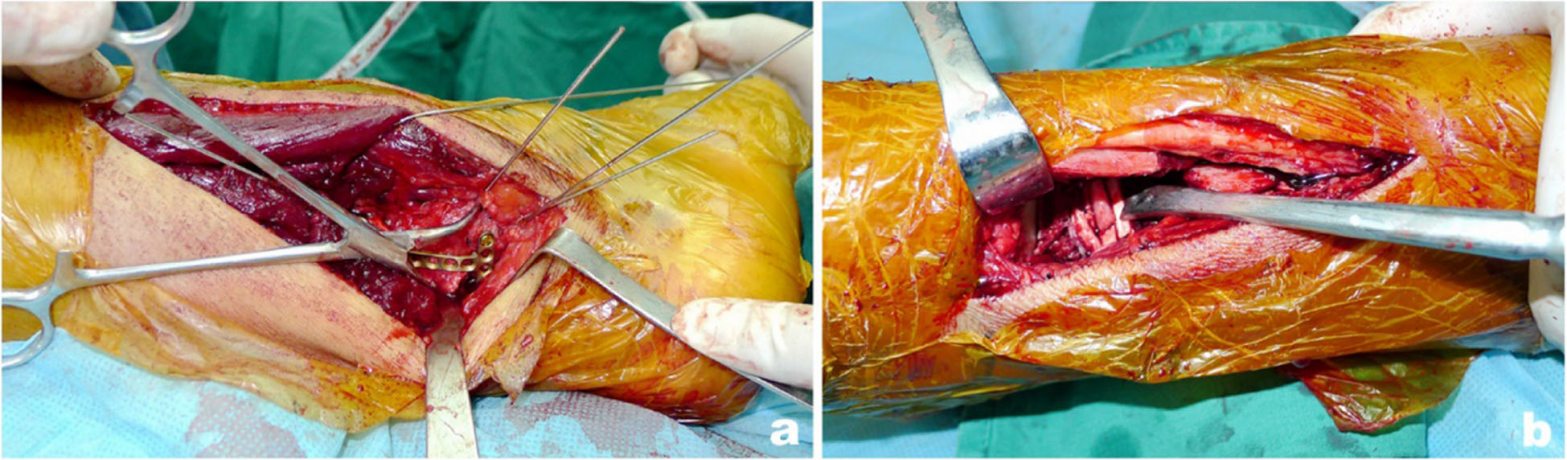
Open reduction was performed 12 days after injury, and fibula and posterrior malleolar fracture (**a**) were fixed through posterolateral approach; the collapsed die-punch fragment (**b**) was reduced by viewing through the anterolateral approach

Patients with right tibial Pilon fractures received preoperative X-ray imaging and CT scans that suggested a collapsed articular surface, freedom of the tarsal tunnel area from the affectation of posterior malleolar fracture fragments, and displacement of the die-punch fragment toward the anteromedial side (Fig. 2). A routinely sterile towel was placed under the lower limb on the affected side, and an airbag tourniquet was applied. A posterolateral approach was first adopted to expose the fibula fracture and perform reduction fixation (Fig. 3). If appropriate, 3.5-mm one-third tubular or reconstruction plates or 3.5-mm locking metaphyseal plates were used for the fixation, although locking plates were used for patients with comminuted fractures and osteoporosis. Then, the posterior malleolar fragment was exposed between the flexor hallucis longus muscle and the peroneus longus and brevis muscles; after reduction, 3.5-mm one-third tubular or locking metaphyseal plates or distal radius locking plates, if appropriate, were applied. The plates were fixed using the unicortical technique. For comminuted fibula fractures, the posterior malleolar fragment was first reduced and fixed, and then a distal tibial anteromedial or anterior approach was adopted, with adjustments as needed according to the specific location of the die-punch fragment, to turn the anterolateral Chaput fragment open, during which special attention was given to protect the attachment points of the anterior talofibular ligament. Using the posterior part of the reduced and fixed articular surface as a reference, the die-punch fragment, the anterolateral Chaput fragment, and the medial malleolar fragment were reduced successively, followed by temporary fixation with Kirschner wires. After C-arm X-ray imaging confirmed satisfactory reduction of the fractures and the articular surface was flattened, the main supporting bone fracture plates were placed on the medial or anterolateral side of the distal tibia along the original displacement direction of the fractures. Miniplates or screws were used as needed to assist in fixation.

#### Posteromedial approach combined with an anterolateral or anterior approach

**Fig. 4:**
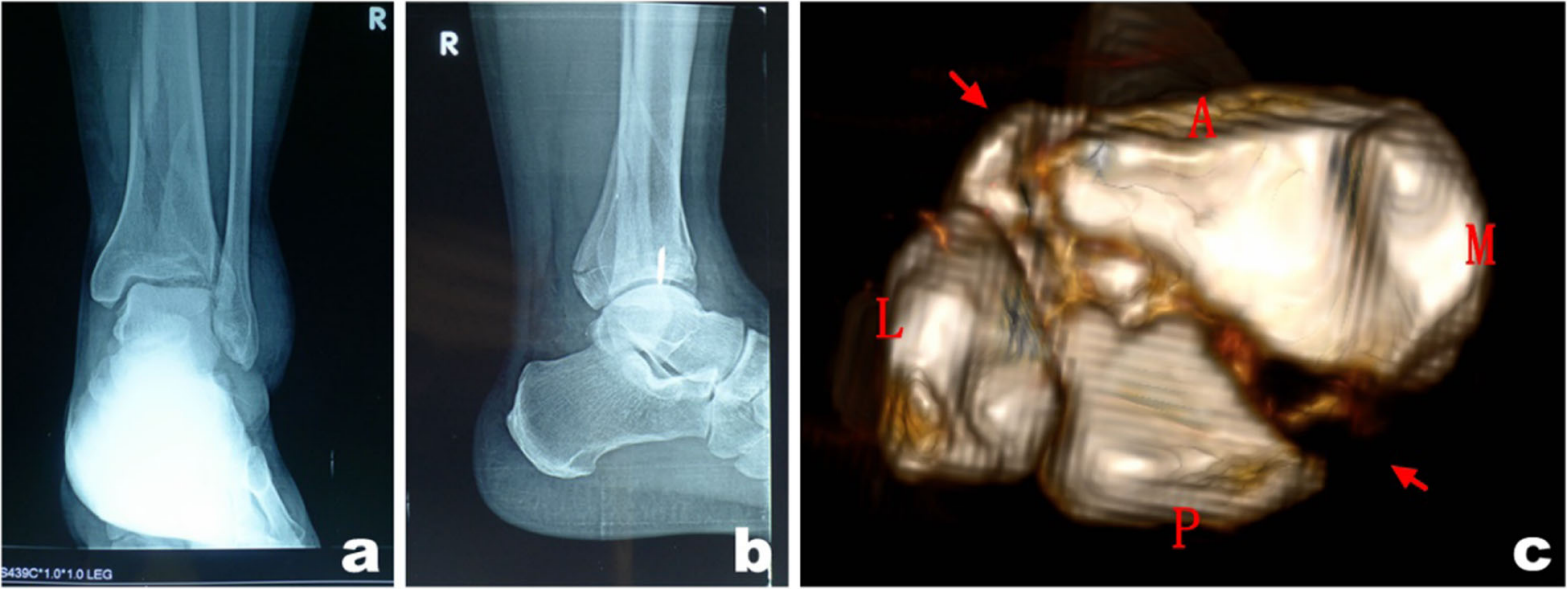
A 36-year-old male patient with Pilon fracture of the right tibia caused by fall injury. Preoperative X-ray (**a**, **b**) and CT scan (**c**) showed that the articular surface collapsed significantly, the posterior malleolar fragment involved the tarsal tunnel area, and the die-punch fragment was slightly anterolateral

**Fig. 5:**
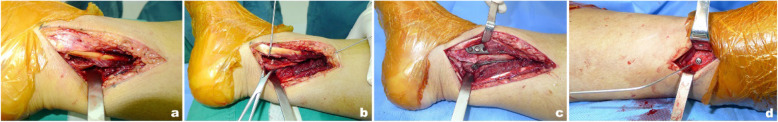
Open reduction was performed 14 days after the injury. Through exploration of the posteromedial approach, the posterior tibial tendon was found to be entrappd between the fracture fragments, and the displaced fracture fragments (**a**–**c**) in the tarsal tunnel area were reduced under direct vision; the Tillaux fracture fragment (**d**) were reduced through the anterolateral approach

Another patient with right tibial Pilon fractures received preoperative X-ray imaging and CT scans that suggested an obviously collapsed articular surface, affectation of posterior malleolar fracture fragments on the tarsal tunnel area, and displacement of the die-punch fragment toward the anterolateral side (Fig. 4). A posteromedial approach was first adopted to expose the tarsal tunnel and loosen the tendons and neurovascular tissues therein (Fig. 5). A retractor was used to retract the tendons and neurovascular tissues for protection, thereby exposing the posterior and medial malleolar fragments. After reduction, 3.5-mm one-third tubular or reconstruction plates, or 3.5-mm locking metaphyseal plates or distal radius locking plates, if appropriate, were used and fixed with the unicortical fixation technique. Then, a distal tibial anterolateral approach was adopted, with adjustments made as needed according to the specific location of the die-punch fragment. Using the posterior part of the reduced and fixed articular surface as a reference, the fragments associated with the collapsed anterolateral articular surface were reduced successively. For comminuted fractures leading to the failure to accurately determine the reduction sign, an anterolateral approach should be adopted first to fix the fibula. After C-arm X-ray imaging confirmed satisfactory reduction of the fractures and the articular surface was flattened, the main supporting bone fracture plates were placed on the medial or anterolateral side of the distal tibia along the original displacement direction of the fractures. Miniplates or screws were used as needed to assist in fixation.

#### Bone grafting and other procedures

After the articular surface reduction, bone defect areas are usually left in the metaphyseal region, which routinely needs to be treated with bone grafting. In order to minimize complications in the donor area, allogenic bone grafting is preferred. In the present study, 5 patients received autologous bone grafting and 25 received allogenic bone grafting. Upon fixation, fluoroscopy was performed again to confirm that the internal fixator had not entered the joint, and ankle mobility was examined to confirm fixation stability. Before closing the incision, absorbable sutures were used to repair the joint capsule and the extensor retinaculum. Should a posteromedial approach be adopted, special attention needs to be paid to the repair of the retinaculum covering the tarsal tunnel in order to prevent complications such as dislocation of the posterior tibial tendon.

### Postoperative management

After all surgeries, a negative pressure drainage tube was placed in the incision, and the ankle joint was fixed at the functional position for 3 weeks using a short leg cast or brace. On the second postoperative day, the patients started passive flexion and extension movements of the toes. After the external fixation was removed, they started ankle flexion and extension exercises. Weight-bearing walking was prohibited until imaging examination confirming the union of the fractures. Generally, weight-bearing walking was started gradually 10–12 weeks after surgery.

### Follow-up and efficacy evaluation

Postoperative patients visited the surgical clinic regularly (1, 3, 6, 12, 18, 24, 36, 48, 60 months after surgery). The quality of fracture reduction was determined using the Burwell-Charnley radiographic criteria, as previously described [7], and patients’ postoperative function was evaluated using the AOFAS Ankle-Hindfoot Scale. The AOFAS Scale gives a score to each item, including pain, gait, ankle functional range, and alignment, with excellent defined as 90–100 points, good as 75–89 points, fair as 50–74 points, and poor as < 50 points.

## Results

The 25 patients (age 39.52 ± 11.37) included in this study were followed for 22–60 months, with an average of 33.9 months (Table 1). Postoperatively, the quality of fracture reduction was determined on a picture archiving and communication system (PACS) workstation according to the Burwell-Charnley radiographic criteria. The results showed that 19 cases had anatomical reduction, 5 had good reduction, and 1 had fair reduction, and that no internal fixator got into the joint. Follow-up imaging examination suggested that the fractures healed within 2–3 months after surgery, with an average healing time of 10.96 weeks. At the last follow-up, no patients were found to have malunion and internal fixation failure. Postoperative function evaluation was performed according to the AOFAS Ankle-Hindfoot Scale. The evaluation results showed that the 25 cases had an average score of 88.4 (range, 80–100), of whom 15 were excellent, 10 good, 0 fair, and 0 poor, with an excellent and good rate of 100%.

**Table 1:** Characteristic of population

Term	Population(*n* = 25)	
	Average (*n*)	SD (%)
Age	39.52	11.37
Gender		
Male	19	76.0%
Female	6	24.0%
Smoking		
Yes	10	40.0%
No	15	60.0%
Side		
Right	14	56.0%
Left	11	44.0%
Follow-up time (months)	33.92	10.40
Injury to OP time (days)	15.96	4.14
Union of fracture (weeks)	10.96	1.43
AOFAS score	88.44	5.01

*AOFAS* American Orthopedic Foot and Ankle Society

**Fig. 6:**
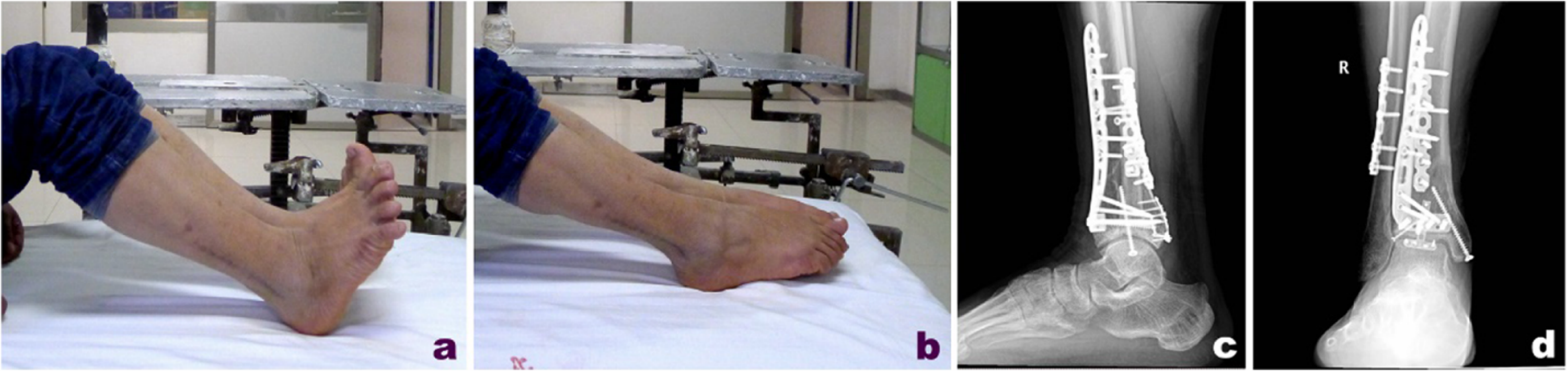
At 36 months follow-up, X-ray reexamination showed that the fracture healed well, no loosening or breakage of the internal fixation were observed, and function recovered well (**a**–**d**)

**Fig. 7:**
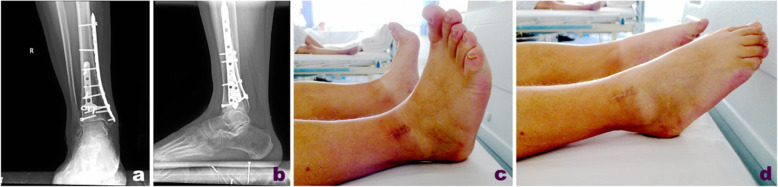
At 48 months follow-up, X-ray reexamination showed that the fracture healed, no loosening or breakage of internal fixation was observed, and function recovered well (**a**–**d**)

For the patients with right Pilon fractures treated with a combined posterolateral and anteromedial approach (tarsal tunnel was not affected by the posterior malleolar fragment), repeat X-ray examination at follow-up 36 months after surgery showed that fractures healed favorably, the internal fixation had no loosening or fracture, and the function recovery was satisfactory (Fig. 6). For patients with right Pilon fractures treated with a combined posteromedial and anterolateral approach (tarsal tunnel was affected by the posterior malleolar fragment), repeat X-ray examination at follow-up 48 months after surgery showed that the fractures had healed, the internal fixation had no loosening or fracture, and the function recovery was satisfactory (Fig. 7).

**Fig. 8:**
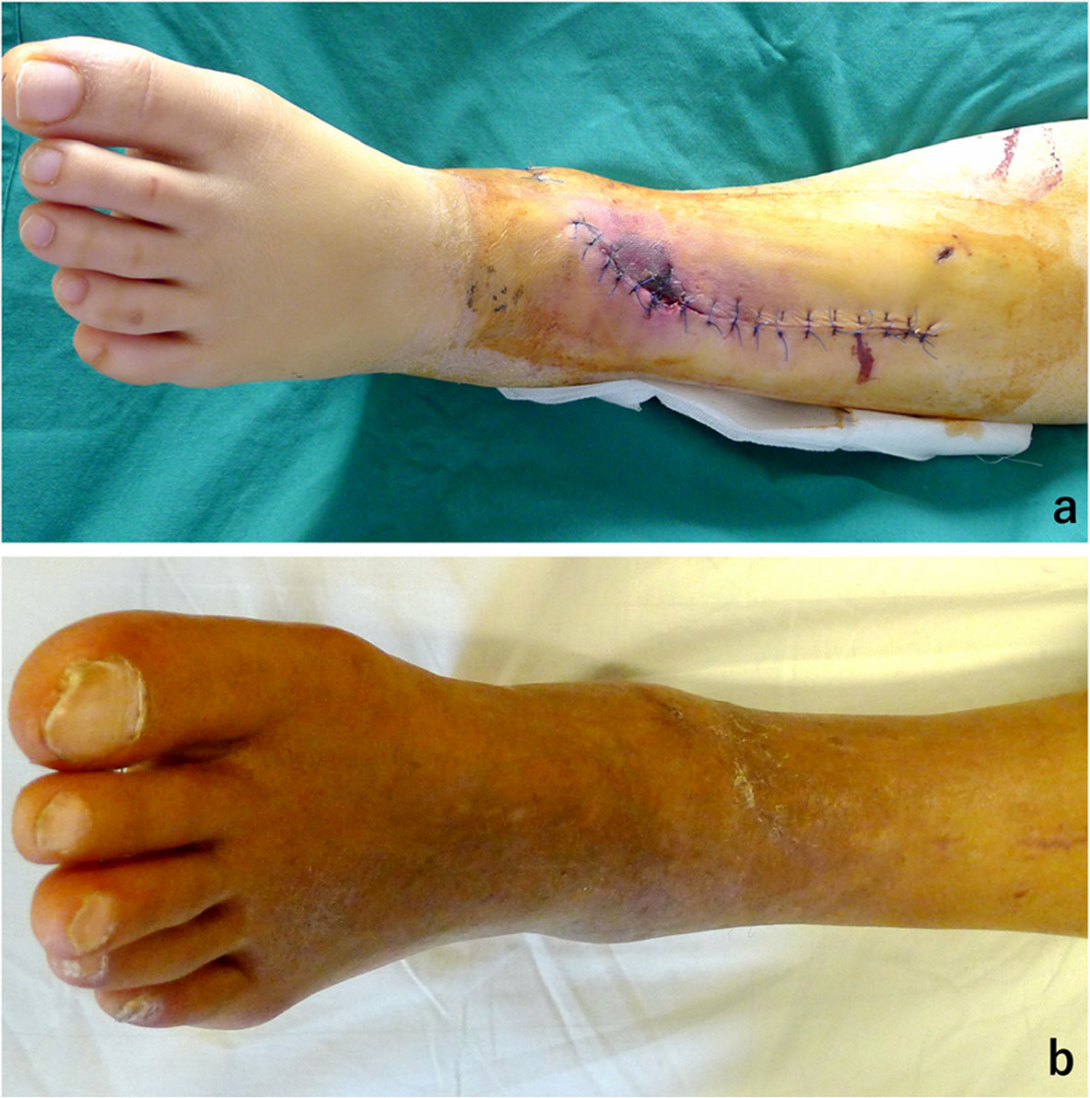
Case18, open reduction was performed 19 days after the injury through the anteromedial with posterolateral approach. Superficial infection of the anteromedial approach was encountered (**a**). This superficial infection was recovered after treatment with local dressing change and oral administration of antibiotics (**b**)

**Fig. 9:**
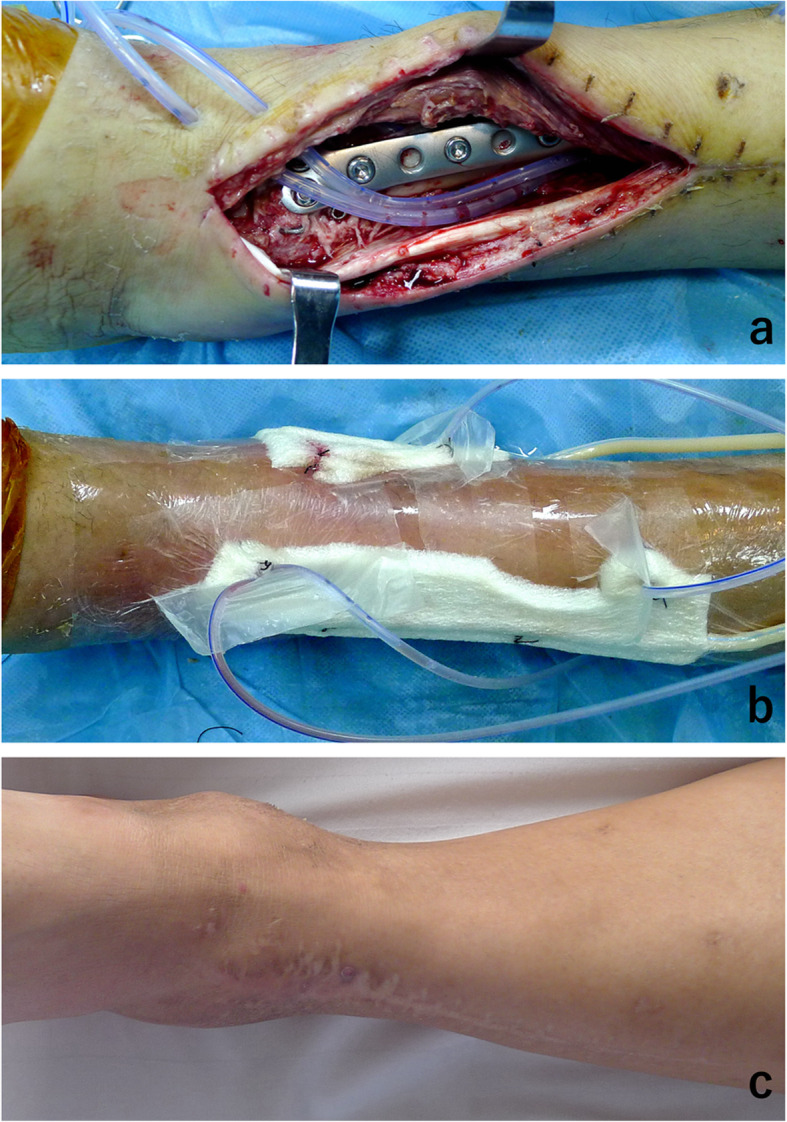
Case24, open reduction was performed 19 days after the injury through the posteromedial with anterolateral approach. Deep infection of anterolateral incision was encountered at 1 week postoperative (**a**), which was controlled by debridement and catheter irrigation performed twice, coupled with intravenous injection of antibiotic (**b**, **c**)

Complications were noted in 7 patients, including osteoarthritis, superficial infection on the anteromedial incision, and delayed union (1 case of type 43C2, 1 case of type 43C3), but recovered after treatment with local dressing change and oral administration of antibiotics (Fig. 8). One patient of type 43C3 had a deep infection on the anterolateral incision, which was controlled by debridement and catheter irrigation performed twice, coupled with intravenous injection of antibiotic (Fig. 9). Two patients treated with a posteromedial surgical approach developed complication of tibial nerve palsy after surgery and recovered after treatment with neurotrophic drugs administered for 3–6 months. No patients treated with a posteromedial surgical approach were observed to have dislocation of the posterior tibial tendon and irritation.

## Discussion

Selecting the most suitable surgical approach to tibia Pilon fractures requires paying special attention to the collapse of the articular surface of the affected area, whether or not the posterior malleolar fragment affects the tarsal tunnel area, and also displacement of the die-punch fragment. The surgical approach strategy adopted by the present study, that is, selecting surgical approaches according to the axial CT scan pattern of the affected area, demonstrated good results in patients’ surgical outcomes. Our findings suggest that CT scans are one of the important clinical bases for the selection of surgical approaches.

By far, the four classic management principles of tibial Pilon fractures and the classic surgical approaches for tibial Pilon fractures proposed by AO/ASIF [2] are still widely followed. However, tibial Pilon fractures vary between cases and each has a certain uniqueness due to differences in injury mechanism. As such, mechanically copying those principles and surgical approaches may lead to unexpected outcomes and even treatment failure in certain severe cases. In fact, according to previous studies, six major surgical approaches can be used for tibial Pilon fractures, namely, medial, anteromedial, anterolateral, lateral, posterolateral, and posteromedial [4, 8], of which the four are most commonly used are the anteromedial, anterolateral, posterolateral, and posteromedial. Each surgical approach has its corresponding exposure range and individual characteristics. The classic anteromedial approach offers good exposure of the anteromedial distal tibia, which favors medial supporting plate placement. However, the approach has an unsatisfactory exposure of the Chaput fragment on the anterolateral side of the distal tibia [4, 9]. Therefore, in order to expand the exposure range of the lateral side of the distal tibia, Assal et al. [3] modified the anteromedial surgical approach. An anterolateral surgical approach offers satisfactory exposure of the anterior side and Chaput fragment of the distal tibia and can also be used to deal with fibular fractures, but has poor exposure of the medial side of the distal tibia [10]. A posterolateral surgical approach can well expose and treat the Volkmann fragment of the distal and posterior fibula, but it fails to offer a direct vision of the ankle articular surface. For this reason, restoration of continuity in the posterior cortex is relied on mainly to indirectly estimate the reduction [11]. This approach is not able to deal with the medial part of the posterior malleolus, so using this approach is not recommended when the posterior malleolar fragment is large (or the tarsal tunnel or the medial malleolus is affected). A posteromedial approach can reveal the medial part of the posterior malleolus, especially the tarsal tunnel. It has irreplaceable advantages for cases in which bone fragment displacement may endanger the posterior tibial vascular nerve and tendon. A standard posteromedial approach goes between the posterior side of the medial malleolus and the Achilles tendon, leading to limited exposure of the lateral part of the posterior malleolus. For this reason, Assal et al. [6] modified the approach, making the incision go along the medial side of the Achilles tendon, which well reveals the entire posterior malleolar fragment and even the posterior side of the lateral malleolus. We have also made corresponding clinical improvements, placing the incision to enter along the posterior side of the medial malleolus. These improvements not only enable us to deal with the posterior malleolus fracture fragment that affects the tarsal tunnel, and to restore the flatness of the bone surface at the tarsal tunnel, but also offer us the vision of the medial malleolar fragment, and even the direct vision of intra-articular fracture in some special cases by turning downward the medial malleolar fragment.

Cole et al. [5] studied the fracture line distribution and the location of main fracture fragments in 38 cases with AO/OTA43C3 comminuted Pilon fractures by CT scan and detected that the main fracture fragments were distributed in the anterolateral, medial, and posterolateral positions. They believed that a clear understanding of fracture morphology contributes to the reasonable choice of surgical approach. In the present study, we examined the affected articular surface of the distal tibial in the included patients by axial CT scans and analyzed their fracture line distribution and bone fragment displacement. Based on the above, the following strategies for surgical approach selection were proposed: when the posterior malleolar fragment was large and affected the tarsal tunnel or the medial malleolus, a posteromedial approach was used to treat the posterior side of the distal tibia, while the anterior side of the distal tibia and fibula fractures was treated with an anterolateral approach. However, when the posterior malleolar fragment was small and did not affect the tarsal tunnel, a posterolateral approach was used to treat the posterior side of the distal tibia and lateral malleolar fractures, while the anterior side of the distal tibia was treated with an anteromedial approach; in this circumstance, the anterior approach is subject to inward or outward adjustment according to the specific position of the die-punch fragment.

In the present study, 24 of 25 patients with complex tibial Pilon fractures treated with surgical approaches selected using the strategies described above achieved excellent fracture reduction and prognosis and had fewer complications. Among the 7 patients treated with the combined posteromedial and anterolateral surgical approach, 3 were found intraoperatively to have the posterior tibial tendon entrapped between the posterior malleolus and the medial malleolus. However, the other 4 patients had the posterior tibial tendon irritated to varying degrees at the posterior malleolar fragment. For these cases, in our opinion, it is inappropriate to adopt conventional surgical approaches that may cause poor reduction and even further damage to the posterior tibial tendon and vascular nerves.

Controversy still exists about whether a combined approach in open reduction internal fixation of closed complex tibial Pilon fractures outperforms the classic surgical approaches proposed by AO/ASIF. Doubts about the anterior-posterior combined approach in internal fixation arise mostly from concerns about the increasing possibility of infections brought about by excessive stripping of soft tissue, delayed union, and nonunion of fractures [12]. To the best of our knowledge, controlled studies comparing the anterior-posterior combined surgical approach with the conventional anterior surgical approach in fixation are lacking. We believe that the application of a surgical approach based on axial CT scans and the implementation of a preoperative plan can reduce unnecessary stripping of soft tissues during surgery and achieve good reduction of articular fragments while protecting soft tissues well. The 25 patients with complete follow-up data in this study all achieved fracture union as expected and were not found to have a significant increase in infection incidence. Although cartilage damage caused at the time of the injury is irreversible, and acts as an important factor that affects later functional recovery, most scholars still agree with the principle that good recovery of the distal tibial articular surface and lower limb alignment, a solid internal fixation, and early functional exercises [8, 13] are important to patients’ outcomes. A combined surgical approach offers good exposure of the distal tibial articular surface, so it leads to a higher probability of obtaining satisfactory fracture reduction. Of the 25 patients who were followed in this study, 24 demonstrated excellent/good fracture reduction. The study of Ketz et al. [14] compared 43C-type tibial Pilon fractures treated with and without posterior fixation and concluded that posterior fixation significantly improved the quality of articular surface reduction. We believe that the anatomical reconstruction of the posterior structure of the distal tibia can transform complex C-type fractures into simpler B-type fractures, thereby simplifying subsequent reduction and fixation operations and also provided a good reference mark for the reduction of the anterior bone fragment. Of note, it is recommended to fix the distal tibial locking plates using a unicortical fixation technique, avoiding affecting the subsequent anterior reduction and fixation operations and facilitating the adjustment of the posterior reduction during surgery when necessary.

The classic treatment principles developed by AO/ASIF recommend that supporting bone plates should be placed on the medial or anterior side of the distal tibia [2]. This recommendation is still adopted by most scholars. However, this principle fails to take into account the possibility of vertical compression violence that causes tibial Pilon fractures to have a valgus or varus tilt. Sirkin et al. [15] found that in the treatment of tibial Pilon fractures caused by valgus violence, supporting bone plates placed on the medial side of the distal tibia performed poorly in countering the trend of fracture displacement, which may lead to fracture *re*-displacement and internal fixation failure. Similar failure cases were also detected in our previous clinical practice. According to our previous research [16], tibial Pilon fractures caused by different mechanisms of valgus and varus injuries lead to inconsistent fracture line distribution and fracture fragment displacement. In view of this, and in order to lower the possibility of internal fixation failure, it is advised that main supporting bone plates be placed in a position that counters the trend of the original displacement of the fracture. In other words, for patients with varus injuries, the main supporting bone plates should be placed on the medial side of the distal tibia, while for patients with valgus injuries, they should be placed on the anterolateral side of the distal tibia; further, for 43C3 type tibial Pilon fractures, multiple bone plates with internal fixation should be used if soft tissue conditions permit, which is expected to create a favorable condition for early postoperative ankle functional exercises. Patients followed in the present study did not have internal fixation failure and malunion, which, in our opinion, should be attributed to compliance with injury mechanisms in placing supporting bone plates.

## Limitations

The present study has a few limitations that must be mentioned, including the small number of included cases, short follow-up time, and lack of a control group, which may cause a certain bias in the research results. In view of these limitations, and in support of results of the present study, large randomized controlled trials are needed to verify the results, with a view toward making the conclusions more scientific and accurate.

## Conclusion

In conclusion, treatment of complex tibial Pilon fractures using surgical approaches selected based on axial CT scans accurately reconstructs the articular surface and achieves solid internal fixation of assembled locking plates, while early postoperative functional exercises contribute to the functional recovery of affected limbs and reduce related complications. This surgical strategy allows surgical approaches and internal fixation methods to be selected specifically based on fracture line distribution on the affected articular surface and bone fragment displacement, reducing soft tissue-related complications and internal fixation failure risk.

## Supplementary information

**Additional file 1: Table S1.** Subject characteristic and injury summary. **Table S2.** Subject treatment summary.

## Data Availability

The datasets analyzed during the current study are available from the corresponding author on reasonable request.

## Abbreviations

AAOS: American Academy of Orthopedic Surgeons (AAOS)
AOFAS: American Orthopedic Foot and Ankle Society
AO/OTA: Association for Osteosynthesis/Orthopedic Trauma Association
AO/ASIF: Association for Osteosynthesis/Association for the Study of Internal Fixation
CT: Computed tomography
