# Supplementary material for: Surgical approach strategies for open reduction internal fixation of closed complex tibial Pilon fractures based on axial CT scans
Source: J Orthop Surg Res. 2020 Jul 27;15:283. doi: 10.1186/s13018-020-01770-y (PMC7385877; doi:10.1186/s13018-020-01770-y)
Supplement: Supplementary file 1 — Additional file 1: Table S1. Subject characteristic and injury summary. Table S2. Subject treatment summary. [file 13018_2020_1770_MOESM1_ESM.docx]

| Supplementary Table S1. Subject characteristic and injury summary. | | | | | | | | |
| --- | --- | --- | --- | --- | --- | --- | --- | --- |
| No. | Age (years) | gender | smoking | side | Means of injury | AO/OTA classification | Injury to surgery (days) |  |
| 1 | 48 | M | Y | L | fall | C1 | 13 |  |
| 2 | 49 | M | N | R | fall | C1 | 21 |  |
| 3 | 46 | M | N | L | car | C1 | 11 |  |
| 4 | 21 | F | N | L | fall | C2 | 20 |  |
| 5 | 42 | M | N | L | fall | C2 | 12 |  |
| 6 | 22 | F | Y | L | fall | C2 | 12 |  |
| 7 | 49 | F | N | L | fall | C3 | 12 |  |
| 8 | 30 | M | N | R | fall | C2 | 15 |  |
| 9 | 30 | M | Y | R | fall | C1 | 17 |  |
| 10 | 30 | M | N | R | fall | C3 | 15 |  |
| 11 | 49 | F | N | R | work | C3 | 24 |  |
| 12 | 47 | M | Y | L | car | C3 | 14 |  |
| 13 | 53 | F | N | R | fall | C3 | 25 |  |
| 14 | 36 | M | N | R | fall | C2 | 10 |  |
| 15 | 53 | M | Y | R | fall | C3 | 18 |  |
| 16 | 24 | M | N | R | fall | C2 | 19 |  |
| 17 | 18 | M | N | L | fall | C2 | 14 |  |
| 18 | 45 | M | Y | R | work | C3 | 19 |  |
| 19 | 40 | F | N | L | fall | C2 | 12 |  |
| 20 | 52 | M | Y | L | fall | C2 | 12 |  |
| 21 | 38 | M | Y | R | fall | C3 | 14 |  |
| 22 | 46 | M | N | R | fall | C3 | 21 |  |
| 23 | 25 | M | Y | R | fall | C3 | 16 |  |
| 24 | 54 | M | Y | L | car | C2 | 14 |  |
| 25 | 41 | M | N | R | work | C3 | 19 |  |
| Abbreviation: M=male, F=female; Y=yes, N=no; R=right, L=left; fall=fall from height; car=car accident, work=work place accident | | | | | | | | |

| Supplementary Table S2. Subject treatment summary. | | | | | | | | |
| --- | --- | --- | --- | --- | --- | --- | --- | --- |
| No. | follow up time (months) | AOFAS score (last follow-up) | Union of fracture (weeks) | Injury mechnical classification | Fracture reduction | Complications | Injury position | Associated injury |
| 1 | 40 | 90 | 8 | varus | anatomic | no | AM, PL | right tibia plateau fracture |
| 2 | 28 | 90 | 10 | neutral | anatomic | no | AM, PL | no |
| 3 | 22 | 90 | 8 | vaglus | anatomic | no | PM, AL | no |
| 4 | 28 | 80 | 12 | varus | anatomic | no | PM, AL | right femur fracture/left talus and calcaneal fracture |
| 5 | 40 | 86 | 12 | varus | anatomic | no | AM, PL | no |
| 6 | 22 | 90 | 12 | varus | good | no | AM, PL | right calcaneal fracture |
| 7 | 28 | 90 | 12 | vaglus | anatomic | no | AM, PL | no |
| 8 | 28 | 80 | 10 | neutral | anatomic | no | AM, PL | no |
| 9 | 28 | 86 | 8 | varus | anatomic | no | AM, PL | no |
| 10 | 44 | 90 | 12 | neutral | good | Delay wound healing | AM, PL | no |
| 11 | 24 | 86 | 12 | varus | pair | no | AM, PL | kidney injury/Rib fracture |
| 12 | 28 | 86 | 12 | neutral | anatomic | no | AM, PL | right tibial fracture |
| 13 | 60 | 90 | 12 | valgus | anatomic | no | PM, AL | no |
| 14 | 34 | 85 | 10 | neutral | anatomic | no | PM, AL | no |
| 15 | 28 | 83 | 12 | neutral | anatomic | no | AM, PL | left calcaneal fracture |
| 16 | 28 | 90 | 12 | vaglus | good | OA | AM, PL | no |
| 17 | 48 | 100 | 10 | vaglus | anatomic | no | AM, PL | no |
| 18 | 48 | 86 | 12 | valgus | anatomic | superficial infection ^a^ | AM, PL | no |
| 19 | 48 | 90 | 10 | varus | anatomic | no | AM, PL | no |
| 20 | 48 | 90 | 12 | neutral | anatomic | Delay wound healing | AM, PL | left patella fracture |
| 21 | 36 | 90 | 12 | valgus | anatomic | OA | AM, PL | no |
| 22 | 28 | 100 | 12 | valgus | good | no | AM, PL | no |
| 23 | 22 | 90 | 12 | valgus | anatomic | no | PM, AL | no |
| 24 | 24 | 93 | 10 | varus | anatomic | infection and skin necrosis of lateral incision ^b^ | PM, AL | right tibia fracture/pelvic fracture |
| 25 | 36 | 80 | 10 | vaglus | good | OA | PM, AL | no |
| Abbreviation: AOFAS=American Orthopaedic Foot and Ankle Society; OA=Osteoarthritis; | | | | | | | | |
| AM=anteromedial; PL=posterolateral; PM=posteromedial; AL=anterolateral; | | | | | | | | |
| ^a^ 12months remove hardware; ^b^ Debridement and skin flap repair | | | | | | | | |
